# 

**DOI:** 10.3109/03009734.2011.597149

**Published:** 2011-06-29

We hope that all our members and readers of the journal had a wonderful summer. Here is some news about the activities of Upsala Medical Society.

After several years of work and negotiations with the Faculty of Medicine, we were able to announce three scholarships for a MD-PhD-program for medical students to support preclinical research. The objectives are to integrate clinical and preclinical research. The due date was May 9 2011.

The Society is also funding purchase of furniture for a Faculty Club in Uppsala Science Park. The room is going to be used for dinners and get-togethers for faculty members with guests.

The annual meeting is going to take place Wednesday September 21. Invitations are going to be sent out soon. This year's honorary lecturer will be Professor Bengt Westermark.

On Friday October 21, the Society and the Disciplinary Domain of Medicine and Pharmacy at Uppsala University are organizing the Olof Rudbeck Day. This year the theme is going to be Cardiovascular Disease with a number of distinguished lecturers. The Rudbeck prize winner of this year will be Professor Lars Wallentin.

Anna Rask-Andersen and Torbjörn Karlsson

